# An Efficient NLOS Errors Mitigation Algorithm for TOA-Based Localization

**DOI:** 10.3390/s20051403

**Published:** 2020-03-04

**Authors:** Yanbin Zou, Huaping Liu

**Affiliations:** 1 Department of Electronic and Information Engineering, Shantou University, Shantou 515063, China; 2 School of Electrical Engineering and Computer Science, Oregon State University, Corvallis, OR 97331, USA; huaping.liu@oregonstate.edu

**Keywords:** cooperative source localization, least squares, non-line-of-sight, semidefinite programming, time-of-arrival

## Abstract

In time-of-arrival (TOA) localization systems, errors caused by non-line-of-sight (NLOS) signal propagation could significantly degrade the location accuracy. Existing works on NLOS error mitigation commonly assume that NLOS error statistics or the TOA measurement noise variances are known. Such information is generally unavailable in practice. The goal of this paper is to develop an NLOS error mitigation scheme without requiring such information. The core of the proposed algorithm is a constrained least-squares optimization, which is converted into a semidefinite programming (SDP) problem that can be easily solved by using the CVX toolbox. This scheme is then extended for cooperative source localization. Additionally, its performance is better than existing schemes for most of the scenarios, which will be validated via extensive simulation.

## 1. Introduction

Localization of an emitting source has a variety of applications, such as emergency assistance, people and asset tracking, location-based advertising and wireless sensor network [1,2,3,4,5]. Measurements used typically include distance-based parameters such as time-of-arrival (TOA) [6,7,8,9] and time-difference-of-arrival (TDOA) [10,11], received signal strength (RSS) [12,13], and angle-of-arrival (AOA) [14].

Some schemes assume line-of-sight (LOS) signal propagation between the source and sensor, or anchor, which is unrealistic for many environments such as indoors. Non-line-of-sight (NLOS) errors could drastically degrade the performance if not taken into consideration in the localization process [15,16].

A straightforward way to mitigate the influence of NLOS errors is identifying and discarding the measurements of the NLOS links [17,18]. However, false-alarm and mis-detection cannot be avoided in this process. Besides, when there are fewer than three remaining nodes for 2D TOA positioning (or four for 3D TOA positioning), the source cannot be localized, but NLOS error mitigation methods are not restricted by the number of LOS nodes [19,20,21,22,23,24,25,26].

In [19], Wang et al. proposed a two-step weighted least squares (2SWLS) method with a linear inequality constraint. The second step of 2SWLS might generate an imaginary source position as a result of taking square-root of a negative quantity. Yang et al. [20] improved the robustness of the scheme in [19] by incorporating the known relationship into the objective function. As in [19], it also ignored the case when the distance measurement noise is negative and path is LOS; the linear inequality constraint cannot be satisfied.

In [21], Vaghefi et al. proposed a semidefinite programming (SDP) method to estimate the source position jointly with the NLOS biases. It requires that the TOA measurement noise variance be known. Besides, in some rare cases, the computation would fail because one of the inequality constraints is not satisfied. Two convex relaxation methods, i.e., the semidefinite relaxation (SDR) and the second-order cone relaxation (SOCR) methods, were developed in [22]. These methods assume a known upper bound about the NLOS errors. The SOCR method also has the convex hull problem, i.e., when the source node is outside the convex hull of the anchor nodes, it cannot provide a good estimate.

In [23], Gao et al. proposed a robust least squares (RLS) method to jointly estimate the source position and the transmission time when address the problem of NLOS error mitigation in the asynchronous sensor network.

Cooperative source localization is known to be effective to improve the accuracy by using the range measurements between the source nodes [27,28], especially when the connectivity in a network is limited and source nodes do not have access to a sufficient number of anchor nodes. However, NLOS mitigation for cooperative source localization is much less studied [15,29].

This paper develops an effective NLOS errors mitigation scheme for TOA-based localization. Different from most of the existing schemes, it requires neither the statistical information of the NLOS errors (e.g., TOA measurement noise variance and NLOS errors upper-bound) nor the identification of NLOS links. Besides, the core of the proposed scheme is an SDP-based algorithm that does not have the convex hull problem. The SDP algorithm is then generalized for cooperative source localization where some links are subject to NLOS errors.

The rest of this paper is organized as follows. Section 2 develops an SDP NLOS error mitigation algorithm for non-cooperative TOA source localization. Section 3 focuses on cooperative source localization where some links are subject to NLOS errors. Section 4 provides simulation results to assess the performance of the proposed scheme.

The following notations are adopted. Bold uppercase and bold lowercase letters denote matrices and vectors, respectively. $∥\cdot ∥$ represents the Euclidean norm. For symmetric matrices A and B, A⪰B means that A−B is positive semidefinite.

## 2. NLOS Error Mitigation Algorithm for Non-Cooperative Source Localization

Consider a wireless sensor network (WSN) with *M* anchor nodes and one source node. Let $s_{i}$ denote the coordinate vector of the *i*th anchor node and u denote the coordinate vector of the source node (for simplicity the development here considers the 2D scenario). Let $d_{i}=∥u-s_{i}∥$ denote the true distance between the *i*th anchor node and the source. The TOA measurements can be written as

(1) $$r_{i}=\left\{\begin{array}{cc} d_{i}+n_{i}+b_{i} & \;i\in N\\ d_{i}+n_{i} & \;i\in L \end{array}\right.$$

where $r_{i}$ is measured range between the *i*th anchor and the source, $\left\{n_{i}\right\}$ are the range measurement noises, which are modeled as independent, zero-mean Gaussian random variables with variance of $\sigma_{i}^{2}$, $b_{i}$ is the NLOS error (positive), and N and L are the indices of the sets corresponding to the NLOS and LOS measurements, respectively. Equation (1) can be written as

(2) $$r_{i}=d_{i}+b_{i}+n_{i},\;i=1,2,\cdots ,M$$

where $b_{i}=0$ for i∈L.

Before the derivation of our proposed algorithm, we briefly review the SDP algorithm in [21]. As in [21], squaring both sides of Equation (2) results in

(3) $$r_{i}^{2}=d_{i}^{2}+2d_{i}b_{i}+b_{i}^{2}+2\left(d_{i}+b_{i}\right)n_{i}+n_{i}^{2}.$$

Letting $h_{i}=d_{i}^{2}$, $q_{i}=2d_{i}b_{i}+b_{i}^{2}$ and $e_{i}=2\left(d_{i}+b_{i}\right)n_{i}+n_{i}^{2}$, Equation (3) can be written as

(4) $$r_{i}^{2}=h_{i}+q_{i}+e_{i}.$$

Finally, Vaghefi et al. gave the following SDP algorithm (corresponding to Equation (19) in [21]):

(5a) $$\begin{array}{cc} \min_{\begin{array}{c} u,y,h_{i},q_{i} \end{array}} & \;\sum_{i=1}^{M}v_{i}{\left(r_{i}^{2}-h_{i}-q_{i}\right)}^{2}+\eta \sum_{i=1}^{M}q_{i}^{2} \end{array}$$

(5b) $$\begin{array}{cc} s.t.\; & h_{i}=y-2u^{T}s_{i}+s_{i}^{T}s_{i} \end{array}$$

(5c) $$\begin{array}{cc} \; & q_{i}\geq 0 \end{array}$$

(5d) $$\begin{array}{cc} \; & \left[\begin{array}{cc} I_{2} & \left(s_{i}-u\right)\\ {\left(s_{i}-u\right)}^{T} & r_{i}^{2}+\mu_{i} \end{array}\right]⪰0_{3} \end{array}$$

(5e) $$\begin{array}{cc} \; & \left[\begin{array}{cc} I_{2} & u\\ u^{T} & y \end{array}\right]⪰0_{3} \end{array}$$

where $v_{i}={\left(r_{i}^{2}\sigma_{i}^{2}\right)}^{-1}$, $\mu_{i}=4r_{i}\sigma_{i}$, and η is a positive penalty factor. We can see that both the weighting element $v_{i}$ and the constraint term $\mu_{i}$ depend on noise variance $\sigma_{i}^{2}$. However, the TOA measurement noise variance $\sigma_{i}^{2}$ is generally unknown in practice [30].

In this paper, we adopt the more realistic assumption that the noise variance is unknown, and then develop an SDP algorithm without sacrificing the performance. The derivation details of proposed algorithm are described next.

The noise term $e_{i}$ can be written as

(6) $$e_{i}=2\left(d_{i}+b_{i}\right)n_{i}+n_{i}^{2}=2\left(r_{i}-n_{i}\right)n_{i}+n_{i}^{2}=2r_{i}n_{i}-n_{i}^{2}.$$

Then $n_{i}$ can be expressed as

(7) $$n_{i}=\frac{e_{i}}{2r_{i}}+\frac{n_{i}^{2}}{2r_{i}}.$$

Equation (2) can be written as

(8) $${\left(r_{i}-n_{i}\right)}^{2}=d_{i}^{2}+2d_{i}b_{i}+b_{i}^{2}=h_{i}+q_{i}.$$

As a result, substituting Equation (7) into Equation (8) yields

(9) $$h_{i}+q_{i}={\left(r_{i}-n_{i}\right)}^{2}={\left(r_{i}-\frac{e_{i}}{2r_{i}}-\frac{n_{i}^{2}}{2r_{i}}\right)}^{2}.$$

Note that

(10) $$r_{i}-\frac{e_{i}}{2r_{i}}\gg \frac{n_{i}^{2}}{2r_{i}}.$$

Thus Equation (9) can be approximated as

(11) $$h_{i}+q_{i}\approx {\left(r_{i}-\frac{e_{i}}{2r_{i}}\right)}^{2}.$$

Furthermore, we use the following convex constraint to relax it:

(12) $$h_{i}+q_{i}\geq {\left(r_{i}-\frac{e_{i}}{2r_{i}}\right)}^{2}.$$

By using Schur complement [31], Equation (12) can be written as

(13) $$\left[\begin{array}{cc} 1 & r_{i}-\frac{e_{i}}{2r_{i}}\\ r_{i}-\frac{e_{i}}{2r_{i}} & h_{i}+q_{i} \end{array}\right]⪰0_{2}.$$

The proposed SDP-based NLOS error mitigation algorithm is expressed as

(14a) $$\begin{array}{cc} \min_{\begin{array}{c} u,y,h_{i},q_{i},e_{i} \end{array}} & \;\sum_{i=1}^{M}e_{i}^{2}+\eta \sum_{i=1}^{M}q_{i}^{2} \end{array}$$

(14b) $$\begin{array}{cc} s.t.\; & r_{i}^{2}=h_{i}+q_{i}+e_{i}, \end{array}$$

(14c) $$\begin{array}{cc} \; & h_{i}=y-2u^{T}s_{i}+s_{i}^{T}s_{i}, \end{array}$$

(14d) $$\begin{array}{cc} \; & q_{i}\geq 0, \end{array}$$

(14e) $$\begin{array}{cc} \; & \left[\begin{array}{cc} 1 & r_{i}-\frac{e_{i}}{2r_{i}}\\ r_{i}-\frac{e_{i}}{2r_{i}} & h_{i}+q_{i} \end{array}\right]⪰0_{2} \end{array}$$

(14f) $$\begin{array}{cc} \; & \left[\begin{array}{cc} I_{2} & u\\ u^{T} & y \end{array}\right]⪰0_{3}. \end{array}$$

In some rare cases (e.g., when the TOA measurement noise $n_{i}$ is large and thus constraint (14e) may not be satisfied), this optimization may be infeasible which can be identified from the cvx status [32]. In such cases, constraint (14e) should be discarded, and algorithm (14) repeated.

Note that the proposed algorithm is different with the algorithm in [21], i.e., Algorithm (5). First, the proposed algorithm does not require any knowledge of the TOA measurement noise variance; second, the convex constraint (14e) is derived and included for improving performance in the proposed algorithm.

## 3. NLOS Error Mitigation Algorithm for Cooperative Source Localization

Now consider a cooperative WSN with *M* anchor nodes (with known positions, $s_{i},i=1,\cdots ,M$) and *N* source nodes (with unknown positions, $x_{i},i=1,\cdots ,N$).

**Figure 1:**
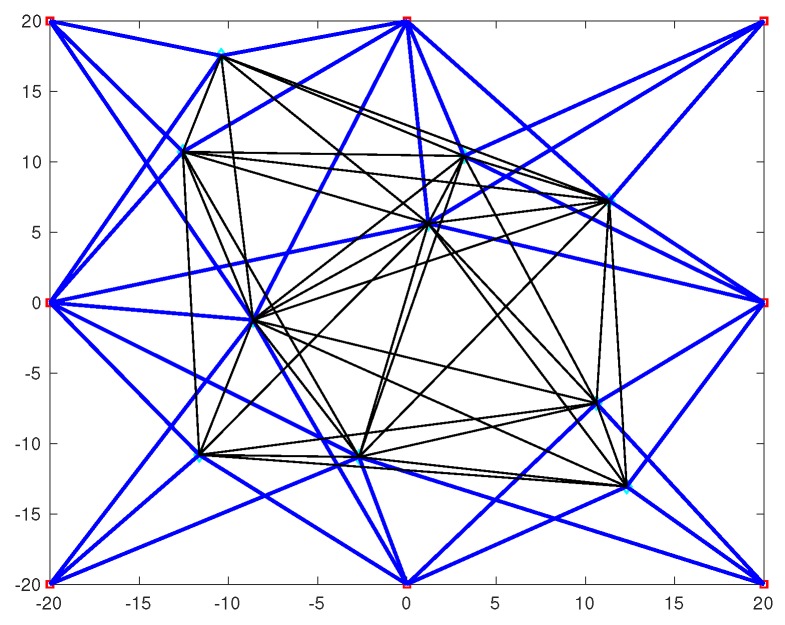
The cooperative source localization geometry of the anchor nodes and source nodes. The eight squares represent the anchor nodes, and the ten diamonds represent the source nodes. The blue lines represent the connections between anchor nodes and source nodes, and the black lines represent the connections between source nodes.

In practice, due to communications range limits, TOA measurements between some node pairs may not be available [27]. Figure 1 gives a geometric illustration for cooperative source localization [33].

Let $d_{0}$ be the maximum communication distance for a possible connection. For n=1,⋯,N, define

(15) $$N\left(n\right)=N_{1}\left(n\right)\cup N_{2}\left(n\right)$$

as the indices of the *n*th source node’s set of neighbors, where

(16a) $$\begin{array}{c} N_{1}\left(n\right)=\left\{k|n<k\leq N,∥x_{n}-x_{k}∥\leq d_{0}\right\}, \end{array}$$

(16b) $$\begin{array}{c} N_{2}\left(n\right)=\left\{m|1\leq m\leq M,∥x_{n}-s_{m}∥\leq d_{0}\right\}. \end{array}$$

This means that a range measurement between source nodes *n* and *k* is available if and only if $k\in N_{1}\left(n\right)$; and a range measurement between source node *n* and anchor node m is available if and only if $m\in N_{2}\left(n\right)$. When NLOS signal propagation is taken into consideration, define the following two kinds of range measurements:

(17) $$f_{nk}=\left\{\begin{array}{cc} ∥x_{n}-x_{k}∥+n_{nk}, & \;\{nk\}\in L\\ ∥x_{n}-x_{k}∥+b_{nk}+n_{nk}, & \;\{nk\}\in N \end{array}\right.$$

and

(18) $$r_{nm}=\left\{\begin{array}{cc} ∥x_{n}-s_{m}∥+w_{nm}, & \;\{nm\}\in L\\ ∥x_{n}-s_{m}∥+c_{nm}+w_{nm}, & \;\{nm\}\in N \end{array}\right.$$

where $n_{mk}$ and $w_{mn}$ are measurement noises, and $b_{mk}$ and $c_{mn}$ are NLOS errors, which are positive and much larger than the measurement noise.

Equation (17) can be rewritten as

(19) $$f_{nk}=v_{nk}+b_{nk}+n_{nk},$$

where $v_{nk}=∥x_{n}-x_{k}∥$ and $b_{nk}=0$ for {nk}∈L. Squaring both sides of Equation (19) yields

(20) $$f_{nk}^{2}=v_{nk}^{2}+2v_{nk}b_{nk}+b_{nk}^{2}+2\left(v_{nk}+b_{nk}\right)n_{nk}+n_{nk}^{2}.$$

Let $g_{nk}=v_{nk}^{2}$, $q_{nk}=2v_{nk}b_{nk}+b_{nk}^{2}$, and $\tau_{nk}=2\left(v_{nk}+b_{nk}\right)n_{nk}+n_{nk}^{2}$. Equation (20) can then be expressed as

(21) $$f_{nk}^{2}=g_{nk}+q_{nk}+\tau_{nk}.$$

Equation (18) can be rewritten as

(22) $$r_{nm}=d_{nm}+c_{nm}+w_{nm},$$

where $d_{nm}=∥x_{n}-s_{m}∥$ and $c_{nm}=0$ for {nm}∈L. Squaring both sides of Equation (22) yields

(23) $$r_{nm}^{2}=d_{nm}^{2}+2d_{nm}c_{nm}+c_{nm}^{2}+2\left(d_{nm}+c_{nm}\right)w_{nm}+w_{nm}^{2}.$$

Let $a_{nm}=d_{nm}^{2}$, $p_{nm}=2d_{nm}c_{nm}+c_{nm}^{2}$, and $\epsilon_{nm}=2\left(d_{nm}+c_{nm}\right)w_{nm}+w_{nm}^{2}$. Equation (23) can then be expressed as

(24) $$r_{nm}^{2}=a_{nm}+p_{nm}+\epsilon_{nm}.$$

We form the following least squares (LS) optimization:

(25a) $$\begin{array}{cc} \min_{\begin{array}{c} x_{n},g_{nk},q_{nk},\tau_{nk},\\ a_{nm},p_{nm},\epsilon_{nm} \end{array}} & \;\sum_{n=1}^{N-1}\sum_{k\in N_{1}\left(n\right)}\tau_{nk}^{2}+\sum_{n=1}^{N}\sum_{m\in N_{2}\left(n\right)}\epsilon_{nm}^{2} \end{array}$$

(25b) $$\begin{array}{cc} s.t.\; & f_{nk}^{2}=g_{nk}+q_{nk}+\tau_{nk}, \end{array}$$

(25c) $$\begin{array}{cc} \; & g_{nk}={∥x_{n}-x_{k}∥}^{2}, \end{array}$$

(25d) $$\begin{array}{cc} \; & q_{nk}\geq 0, \end{array}$$

(25e) $$\begin{array}{cc} \; & r_{nm}^{2}=a_{nm}+p_{nm}+\epsilon_{nm}, \end{array}$$

(25f) $$\begin{array}{cc} \; & a_{nm}={∥x_{n}-s_{m}∥}^{2}, \end{array}$$

(25g) $$\begin{array}{cc} \; & p_{nm}\geq 0. \end{array}$$

Let $X=[x_{1},\cdots ,x_{N}]$ and $Y=X^{T}X$. Equations (25c) and (25f) can be rewritten as

(26) $$g_{nk}=Y\left(n,n\right)-2Y\left(n,k\right)+Y\left(k,k\right)$$

and

(27) $$a_{nm}=Y\left(n,n\right)-2x_{n}^{T}s_{m}+s_{m}^{T}s_{m}.$$

The non-convex constraint $Y=X^{T}X$ can be relaxed as

(28) $$\left[\begin{array}{cc} I_{2} & X\\ X^{T} & Y \end{array}\right]⪰0_{N+2}.$$

Similar to the derivation of (13), we obtain the following convex constraints

(29) $$\left[\begin{array}{cc} 1 & f_{nk}-\frac{\tau_{nk}}{2f_{nk}}\\ f_{nk}-\frac{\tau_{nk}}{2f_{nk}} & g_{nk}+q_{nk} \end{array}\right]⪰0_{2}$$

and

(30) $$\left[\begin{array}{cc} 1 & r_{nm}-\frac{\epsilon_{nm}}{2r_{nm}}\\ r_{nm}-\frac{\epsilon_{nm}}{2r_{nm}} & a_{nm}+p_{nm} \end{array}\right]⪰0_{2}.$$

Here two penalty terms $\eta \sum_{n=1}^{N-1}\sum_{k\in N_{1}\left(n\right)}q_{nk}^{2}$ and $\eta \sum_{n=1}^{N}\sum_{m\in N_{2}\left(n\right)}p_{nm}^{2}$ are also needed in the objective function to ensure that the values of $q_{nk}$ and $p_{nm}$ are within a reasonable range.

The above analysis finally leads to the following NLOS error mitigation algorithm for cooperative localization:

(31a) $$\begin{array}{cc} \min_{\begin{array}{c} X,Y,\\ g_{nk},q_{nk},\tau_{nk},\\ a_{nm},p_{nm},\epsilon_{nm} \end{array}} & \;\sum_{n=1}^{N-1}\sum_{k\in N_{1}\left(n\right)}\left(\tau_{nk}^{2}+\eta q_{nk}^{2}\right)+\sum_{n=1}^{N}\sum_{m\in N_{2}\left(n\right)}\left(\epsilon_{nm}^{2}+\eta p_{nm}^{2}\right) \end{array}$$

(31b) $$\begin{array}{cc} s.t.\; & f_{nk}^{2}=g_{nk}+q_{nk}+\tau_{nk}, \end{array}$$

(31c) $$\begin{array}{cc} \; & g_{nk}={∥x_{n}-x_{k}∥}^{2}, \end{array}$$

(31d) $$\begin{array}{cc} \; & q_{nk}\geq 0, \end{array}$$

(31e) $$\begin{array}{cc} \; & r_{nm}^{2}=a_{nm}+p_{nm}+\epsilon_{nm}, \end{array}$$

(31f) $$\begin{array}{cc} \; & a_{nm}={∥x_{n}-s_{m}∥}^{2}, \end{array}$$

(31g) $$\begin{array}{cc} \; & p_{nm}\geq 0, \end{array}$$

(31h) $$\begin{array}{cc} \; & \left[\begin{array}{cc} 1 & f_{nk}-\frac{\tau_{nk}}{2f_{nk}}\\ f_{nk}-\frac{\tau_{nk}}{2f_{nk}} & g_{nk}+q_{nk} \end{array}\right]⪰0_{2} \end{array}$$

(31i) $$\begin{array}{cc} \; & \left[\begin{array}{cc} 1 & r_{nm}-\frac{\epsilon_{nm}}{2fr_{nm}}\\ r_{nm}-\frac{\epsilon_{nm}}{2r_{nm}} & a_{nm}+p_{nm} \end{array}\right]⪰0_{2} \end{array}$$

(31j) $$\begin{array}{cc} \; & \left[\begin{array}{cc} I_{2} & X\\ X^{T} & Y \end{array}\right]⪰0_{N+2}. \end{array}$$

Like the algorithm for non-cooperative source localization, the above SDP algorithm might be infeasible in some rare cases. Nevertheless, discarding the constraints (31h) and (31i), the proposed algorithm still provides good estimation. The derived constraints (31h) and (31i) aim to improve the estimation accuracy.

## 4. Simulation Results

This section provides extensive numerical simulations to assess the performance of the proposed algorithm. Comparisons are made with existing NLOS error mitigation algorithms: [19] (labeled as LS), [20] (labeled as QP), [21] (labeled as SDP-Reza), and [22] (labeled as SDP-Robust, corresponding to (56) in [22]). Note that for SDP-Reza, computation would fail because constraint (5d) is not satisfied. In such cases, discard constraint (5d) and repeat the algorithm.

For cooperative source localization, the proposed algorithm is compared with [29] (labeled as Reza) as well as the maximum likelihood (ML) algorithm (label as ML, it assumes all the links are LOS).

The proposed estimator and other convex relaxation algorithms are implemented in CVX [32] using SeDuMi as a solver [34] with precision set to ‘best’. The root mean-square error (RMSE) is chosen as the performance metric for non-cooperative source localization, which is defined as $\mathrm{RMSE}=\sqrt{\frac{1}{K}\sum_{j=1}^{K}{∥{\hat{u}}_{j}-u∥}^{2}}$, where ${\hat{u}}_{j}$ is the estimate of the source position in the *j*th run and *K* is the number of Monte Carlo runs. For cooperative source localization, the performance matric is the average root mean-square error (ARMSE), defined by $\mathrm{ARMSE}=\sqrt{\frac{1}{NK}\sum_{j=1}^{K}\sum_{n=1}^{N}{∥{\hat{x}}_{n}^{j}-x_{n}∥}^{2}}$, where ${\hat{x}}_{n}^{j}$ is the estimate of the *n*th source position in the *j*th run.

### 4.1. Non-Cooperative Localization

The simulation for non-cooperative localization here assumes a wireless sensor network with one source and eight sensors located at ${\left[20,20\right]}^{T}$ m, ${\left[20,-20\right]}^{T}$ m, ${\left[-20,20\right]}^{T}$ m, ${\left[-20,-20\right]}^{T}$ m, ${\left[20,0\right]}^{T}$ m, ${\left[-20,0\right]}^{T}$ m, ${\left[0,20\right]}^{T}$ m, ${\left[0,-20\right]}^{T}$ m. The position of the source is randomly generated and uniformly distributed over the area of [−30,30]×[−30,30] m${}^{2}$. The range measurement noise $n_{i}$ follows the Gaussian distribution with zero-mean and variance $\sigma^{2}$ and NLOS error $b_{i}$ is uniformly distributed over $\left[0,B_{\max}\right]$ [22]. The penalty factor is set to η=0.01. Each simulation generates 1000 Monte Carlo realizations.

**Figure 2:**
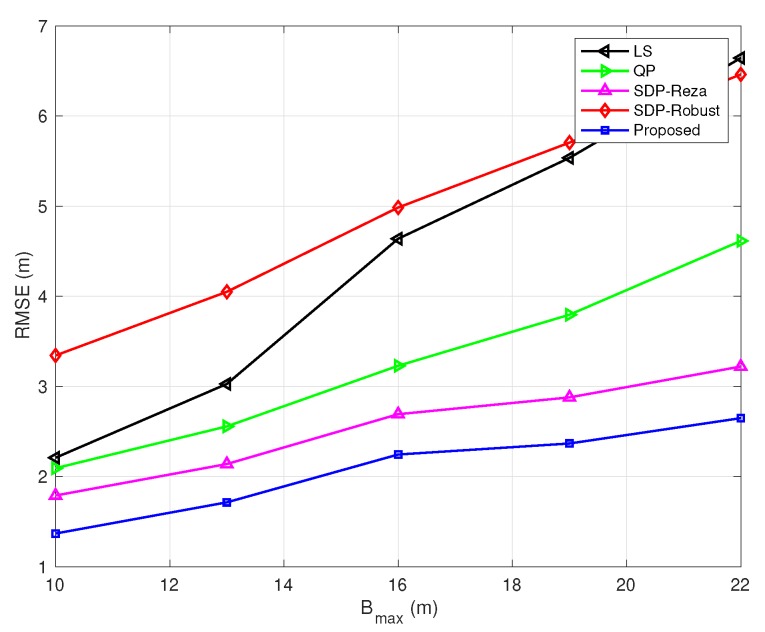
Root mean-square errors (RMSEs) vs. non-line-of-sight (NLOS) error bound $B_{\max}$ (σ=0.5 m, and the number of NLOS links equals 4).

In Figure 2, the TOA measurement noise variance and the number of NLOS links are fixed; the NLOS error bound $B_{\max}$ varies from 10 m to 22 m. The RMSE of all the methods increases as $B_{\max}$ increases, as expected. In terms of relative performance, the proposed algorithm provides the best performance, then the SDP-Reza algorithm, and then the QP and LS algorithms, while the SDP-Robust algorithm performs the worst.

**Figure 3:**
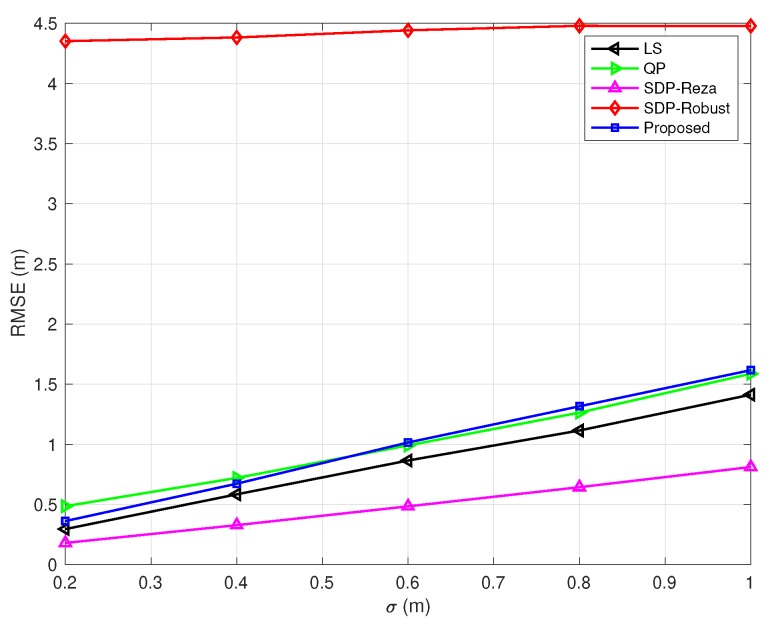
RMSEs vs. σ $(B_{\max}=10$ m and the number of NLOS links equals 0).

**Figure 4:**
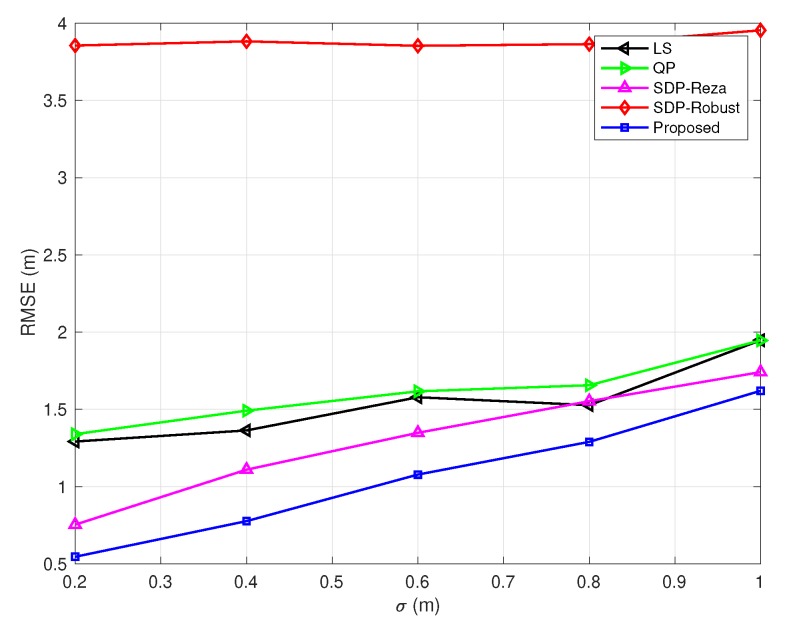
RMSEs vs. σ $(B_{\max}=10$ m and the number of NLOS links equals 2).

**Figure 5:**
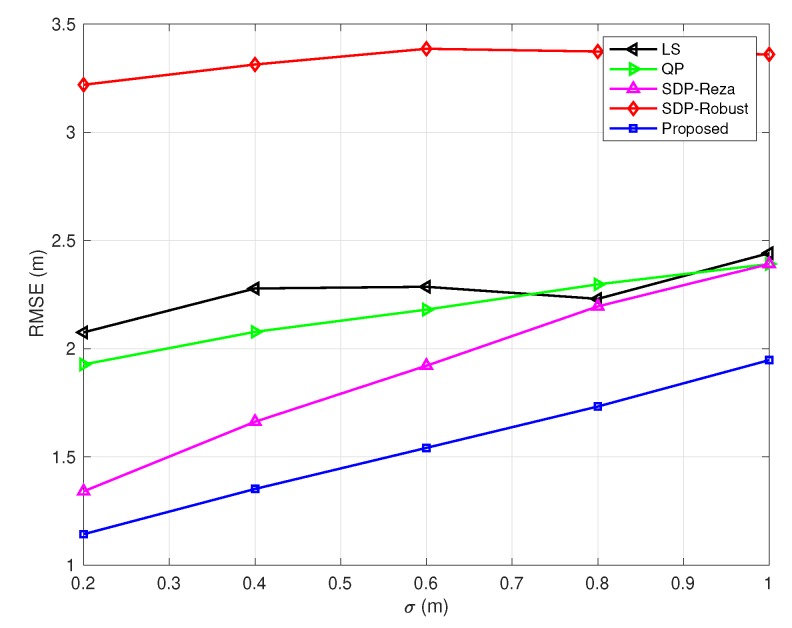
RMSEs vs. σ $(B_{\max}=10$ m and the number of NLOS links equals 4).

**Figure 6:**
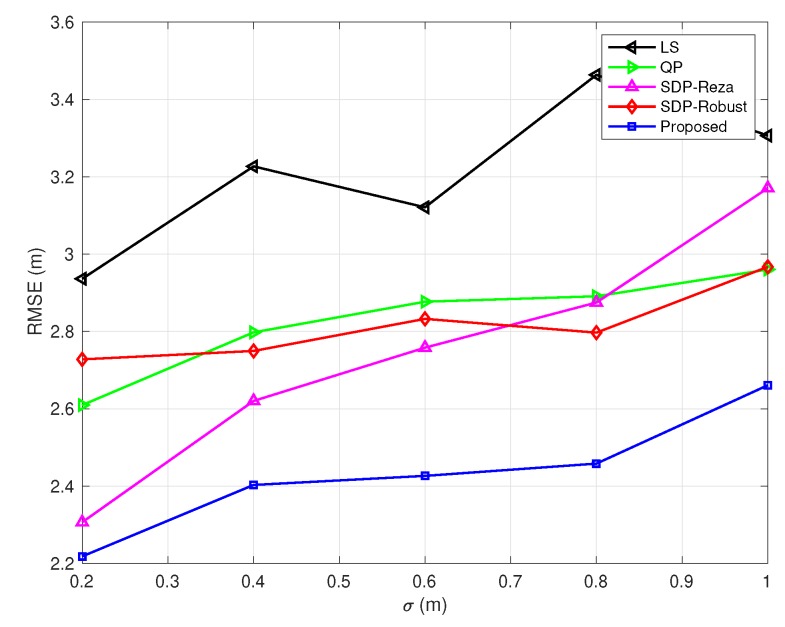
RMSEs vs. σ $(B_{\max}=10$ m and the number of NLOS links equals 6).

**Figure 7:**
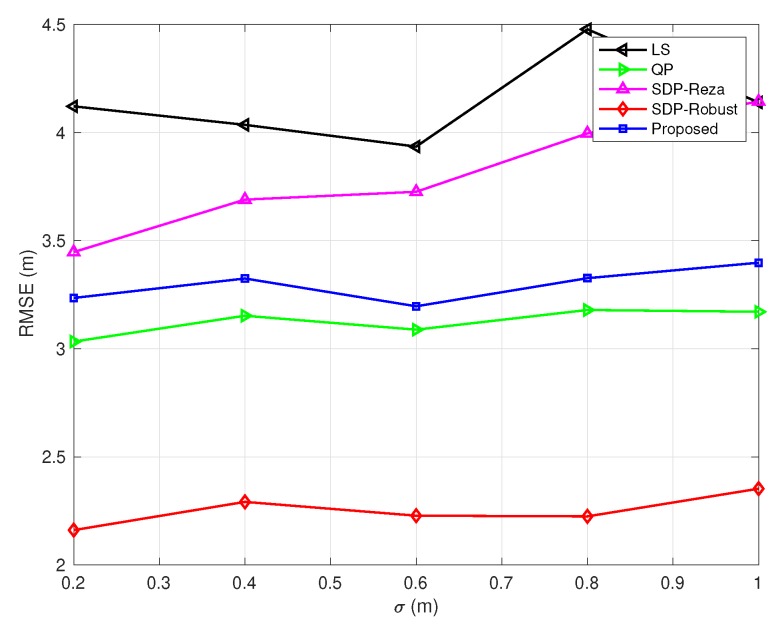
RMSEs vs. σ $(B_{\max}=10$ m and the number of NLOS links equals 8).

In Figure 3, Figure 4, Figure 5, Figure 6 and Figure 7, the NLOS error bound is fixed, while the number of NLOS links varies from 0 to 8 and the standard deviation σ varies from 0.2 m to 1 m. Figure 3 shows the case with all LOS links, and Figure 7 shows the case with all NLOS links. These figures lead to three observations: (1) the SDP-Reza algorithm has the best performance when all the links are LOS; this is because its weighting element is accurate in this case. (2) The SDP-Robust algorithm has the best performance when all the links are NLOS, because it assumes all the links are NLOS. (3) The proposed algorithm has the best performance when the number of NLOS links equal to 2, 4, 6, respectively.

It should be noted that the proposed algorithm cannot provide the best performance when all the links are LOS or NLOS. Except for the two extreme cases, the proposed algorithm can give the best performance. In practice, the number of NLOS links is unknown. The majority of scenarios are LOS/NLOS mixed, and the proposed algorithm can provide best performance in these scenarios.

### 4.2. Cooperative Source Localization

For cooperative source localization, a WSN with eight anchors and ten sources, as showing in Figure 1 is considered. The positions of anchor nodes are the same as the non-cooperative case above. The positions of the source, as discussed earlier, are randomly generated, and once realization outcome is (−8.6237,−1.2310)m, (−10.4088,17.5334)m, (12.3117,−13.0601)m, (10.6205,−7.1419)m, (−2.6837,−10.9620)m, (3.1923,10.4146)m, (1.1929,5.6211)m, (−11.6372,−10.8073)m, (11.3331,7.2338)m and (−12.5562,10.7131)m [33]. The range measurement noise $n_{nk}$ and $w_{nm}$ both follow the Gaussian distribution with zero-mean and variance $\sigma^{2}$. The NLOS error $b_{nk}$ and $c_{nm}$ both follow the uniform distribution in $\left[0,B_{\max}\right]$. The penalty factor is set to η=0.01. The maximum communication distance is 25 m. The results are obtained from 1000 independent realizations.

**Figure 8:**
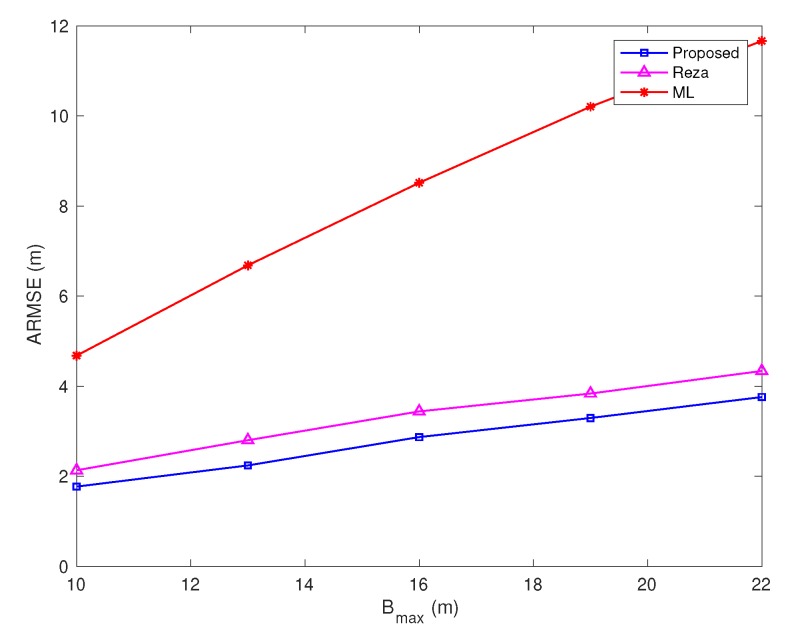
Average root mean-square errors (ARMSEs) vs. NLOS error bound $B_{\max}$ (σ=0.5 m, and the percentage of NLOS links equals 50%).

In Figure 8, the variance of TOA measurement noise and the percentage of NLOS links are fixed (the number of NLOS links is the floor function of the percentage times all links), while the NLOS error bound $B_{\max}$ varies from 10 m to 22 m. As expected, the ARMSE of three methods increases as $B_{\max}$ increases, but the proposed algorithm clearly better than the Reza algorithm and ML algorithm. The ML algorithm provides the worst performance, because it assumes all the links are LOS. The ARMSE of proposed algorithm is about 0.7 m less than the Reza algorithm when $B_{max}=22$ m.

**Figure 9:**
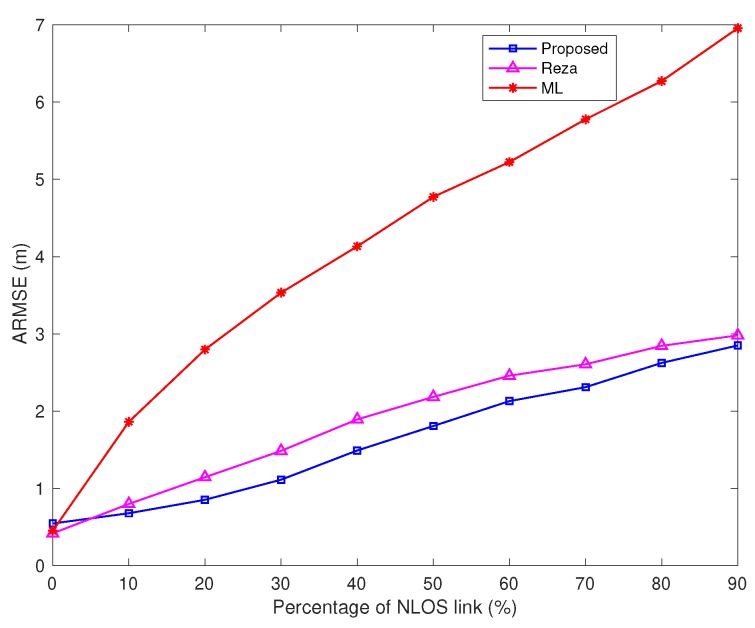
ARMSEs vs. percentage of NLOS links (σ=0.5 m, and the NLOS error bound $B_{\max}$ is 10 m).

In Figure 9, the variance of TOA measurement noise and the NLOS error bound are fixed, while the percentage of NLOS links varies from 0 to 90%. For this case, the proposed algorithm still better than Reza algorithm except for one scenario: all the links are LOS. The ARMSE of ML algorithm increases quickly as the percentage of NLOS links increases.

**Figure 10:**
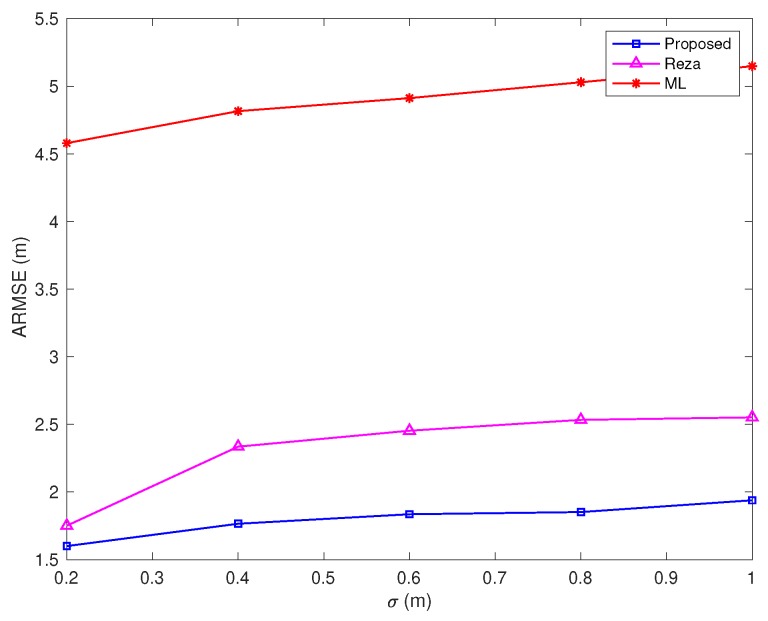
ARMSEs vs. σ ($B_{\max}=10$ m, and the percentage of NLOS links equals 50%).

In Figure 10, the NLOS error bound and the percentage of NLOS links are fixed, while the standard deviation σ varies from 0.2 m to 1 m. As in the previous case, the proposed algorithm still performs well.

## 5. Conclusions

An effective scheme is proposed for source localization with TOA measurements, some of which are subject to NLOS errors. It is an SDP based algorithm, the details of which are first developed for non-cooperative localization. It is then generalized to the case of cooperative source localization where some of the TOA measurements are subject to NLOS errors. Unlike most of the existing related work, the proposed scheme does not need to know the statistical information of the NLOS errors or information about the TOA measurement noise variance. It also performs better than existing schemes for the majority of the scenarios evaluated except a few isolated cases.

## Abbreviations

The following abbreviations are used in this manuscript:

TOA	Time-of-arrival
NLOS	Non-line-of-sight
SDP	Semidefinite programming
TDOA	Time-difference-of-arrival
RSS	Received signal strength
AOA	angle-of-arrival
2SWLS	Two-step weighted least squares
SDR	Semidefinite relaxation
SOCR	Second-order cone relaxation
LOS	Line-of-sight
RMSE	Root mean-square error
ARMSE	Average root mean-square error
ML	Maximum likelihood
